# SARS-CoV2 infects pancreatic beta cells in vivo and induces cellular and subcellular disruptions that reflect beta cell dysfunction

**DOI:** 10.21203/rs.3.rs-592374/v1

**Published:** 2021-07-20

**Authors:** Katelyn Millette, Janielle Cuala, Peiyu Wang, Carolyn Marks, Veronica Woo, Maya Hayun, Harsimar Kang, Martin Martin, Sangeeta Dhawan, Lily Chao, Scott Fraser, Jason Junge, Mark Lewis, Senta Georgia

**Affiliations:** USC; USC; USC; USC; USC; USC; USC; UCLA; City of Hope; CHLA; USC; University of Southern California; Bioqual; Children’s Hospital Los Angeles

**Keywords:** COVID-19, SARS-CoV2, Diabetes, Type 2 Diabetes, Beta cell Injury, Non-human primates

## Abstract

Increasing evidence of new-onset diabetes during the COVID19 pandemic indicates that the SARS-CoV2 virus may drive beta-cell dysfunction leading to diabetes, but it is unclear if it is a primary or secondary effect. Here, we present evidence of SARS-CoV-2 infection of pancreatic beta cells *in vivo* using a robust and reproducible non-human primates model of mild to moderate COVID19 pathogenesis. Pancreas from SARS-CoV-2 infected subjects were positive for the SARS-CoV2 spike protein by immunohistochemistry and structures indicative of viral replication were evident by electron microscopy. Total beta cell area was decreased in SARS-CoV-2-infected pancreas, attributable to beta cell atrophy. Beta cell granularity was decreased. These histologic phenotypes persisted beyond the duration of the clinical disease course. Detailed electron microscopy of SARS-CoV-2 infected beta-cells revealed ultrastructural hallmarks of beta cell stress that are seen in islets of patients with Type 2 diabetes, including disrupted mitochondria and dilated endoplasmic reticulum. To assess the metabolic status of beta cells from SARS-CoV-2-infected subjects, we used fluorescence life-time imaging to measure the ratio of free and bound NADH as a surrogate of glycolytic and oxidative metabolism. We report an increase in free NADH levels, suggesting that beta cells from SARS-CoV-2-infected subjects adopt a more glycolytic metabolic profile. Taken together, we conclude that SARS-CoV-2 infection induces beta cell stress that may compromise beta-cell function beyond the duration of the disease course. This raises the possibility that the beta cell stress and injury may have clinical implications of the long-term future health of patients that have recovered from COVID19.

## Introduction

Since the beginning of the SARS-CoV-2 pandemic, there has been a concern about the possibility of infection precipitating the new onset diabetes (1–5). It has been postulated that there may be a bidirectional relationship between COVID19 and diabetes, but it is unclear if that relationship can be directly attributed to loss of beta cell function after SARS-CoV-2 infection of cells, or due to indirect beta cell stress from increased insulin resistance, steroid treatment, and global inflammation. Several high profile reports have provided conflicting results about the presence of ACE2, TMPRSS2, and SARS-CoV-2 in the beta cells of infected patients (6–10). While these reports have been very informative, they are limited by the difficulty in acquiring patient tissue and that the available post-mortem tissues are limited to patients who have expired due to severe illness. Because most illness from SARS-CoV-2 infection is not severe, it is critical to identify a reproducible model system to study the effects of SARS-CoV-2 infection on beta cells during and after the disease course.

During the SARS-CoV-2 pandemic, non-human primates have proven to be a consistent, robust, and reproducible model for studying COVID19 disease pathophysiology and for preclinical evaluation of vaccines and therapeutics (11–25). It has been reported that rhesus macaques infected intranasally/intratracheally with SARS-CoV-2 show a mild to moderate disease pathology consistent with the overwhelming majority of human COVID19 cases. We capitalized on our access to this model system to evaluate the effects of mild/moderate COVID19 disease pathogenesis on beta cells *in vivo*. We obtained the pancreas from adult Rhesus Macaques inoculated with SARS-CoV-2 and of uninfected adult macaques collected at necropsy. We evaluated cellular histology, subcellular ultrastructure, and metabolic signatures to assess if SARS-CoV-2 infected beta cells *in vivo* and whether SARS-CoV-2 infection resulted in aberrant cellular pathology characteristic of functional beta cell impairment.

### SARS-COV-2 infects the beta cells of NHP *in vivo*

As previously reported, adult rhesus macaques (6 to 12 years of age) were inoculated with 1.1 × 10^6^ plaque-forming units (PFU) of SARS-COV-2 administered as 1 ml intranasally and 1 ml intratracheally (13, 15, 16). In this model, viral RNA levels peak at 2 days post inoculation (dpi). Interstitial viral pneumonia is present and resolves by around 4 dpi. Clinical disease course is resolved by around 12dpi (16). To assess if there was an acute effect of SARS-COoV-2 infection on beta cells and if it resolved by the end of the disease course, we evaluated the pancreas collected at necropsy during the acute phase (7–10dpi, n=3) and in the post-acute phase (14dpi, n=4) of the disease course. The pancreas from 3 uninfected adult macaques and 2 pregnant macaques infected with the Zika virus served as controls (26).

Because there have been conflicting reports about the robustness of *ACE2* and *TMPRSS2* expression in human islets, we sought to identify if either transcript was present in the islets of rhesus macaques. We interrogated a publicly available single cell sequencing data set from multiple organs of the rhesus macaque (27). Cells were clustered into organ specific clusters, and the both *ACE2* and *TMPRSS2* were present in the pancreas cluster. We then clustered cells into pancreatic cell subtypes. *ACE2* and *TMPRSS2* expression was highest in beta cells (Supplemental Figure 1A). We confirmed ACE2 expression in beta cells by immunohistochemistry. ACE2 expression was low but present in most beta cells; a subset of cells exhibited robust expression (white arrows, Supplemental Figure 1B).

**Figure 1:**
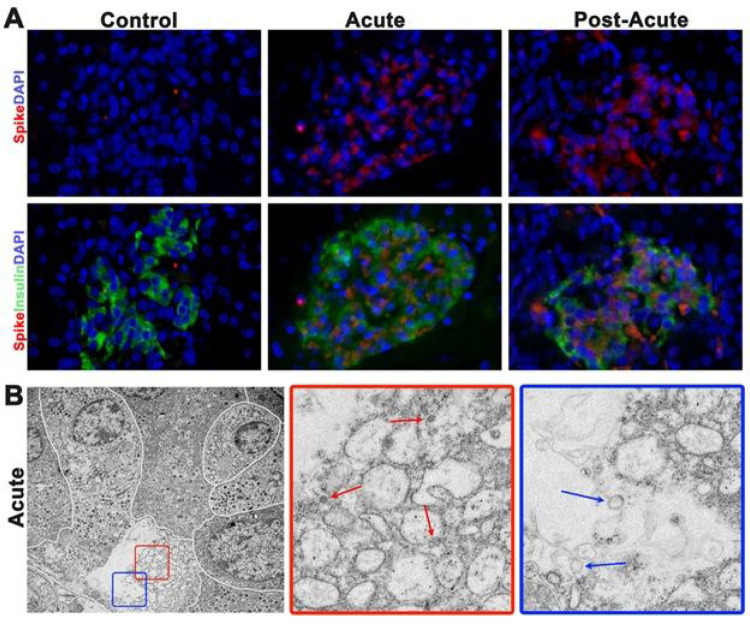
SARS-CoV-2 directly infects beta-cells in vivo. (A) Representative images of immunofluorescent staining for SARS-CoV-2 nucleocapsid protein (red) and insulin (green) in control and inoculated NHPs. SARS-CoV-2 nucleocapsid protein was not detected in control tissues. SARS-CoV-2 protein is not present in every beta cell and it is also present in non-beta islet cells. (B) Representative transmission electron microscopy of an islet from an acute subject. White lines delineate cell borders. Less-electron dense beta cell has distended borders and encompasses a viral replication complex. Red and blue box insets are high magnification of selected areas. Note viral particles at various stages of maturity inside of the vacuoles of the replication complex (red arrows). Double membraned vacuoles are also hallmarks of viral replication (blue arrows).

After establishing that *ACE2* was present in the beta cells of this system, we used immunohistochemistry to detect the SARS-COV-2 nucleocapsid protein in the islets of acute or post-acute pancreas. Because SARS-CoV2 RNA is no longer detectable in the bronchioalveolar fluid of post-acute subjects by Day 14, it was unclear if the infection would be present only during the acute phase or would resolve by the post-acute phase (16). Islets from acute and post-acute pancreas were positive for the nucleocapsid protein (Figure 1A). Controls tissues were negative for nucleocapsid protein expression. To confirm the presence of the virus in acute and post-acute islet cells, we used transmission electron microscopy to assess if viral particles were present in beta cells from inoculated subjects. Beta cells were identified by the characteristic halos around secretory granules. Viral particles were present in 4 of the 4 samples assessed by electron microscopy. An active viral replication complex was also present and contained structures that were representative of multiple stages of viral particle assembly (Figure 1B, red box and red arrows) (28). We noted characteristic double membrane vesicles inside of the profusion replication complex are also hallmarks of SARS-CoV2 infection we also present (Figure 1B, blue box and blue arrows) (29).

### SARS-CoV2 infection drives a massive loss of beta cell mass

**Figure 2:**
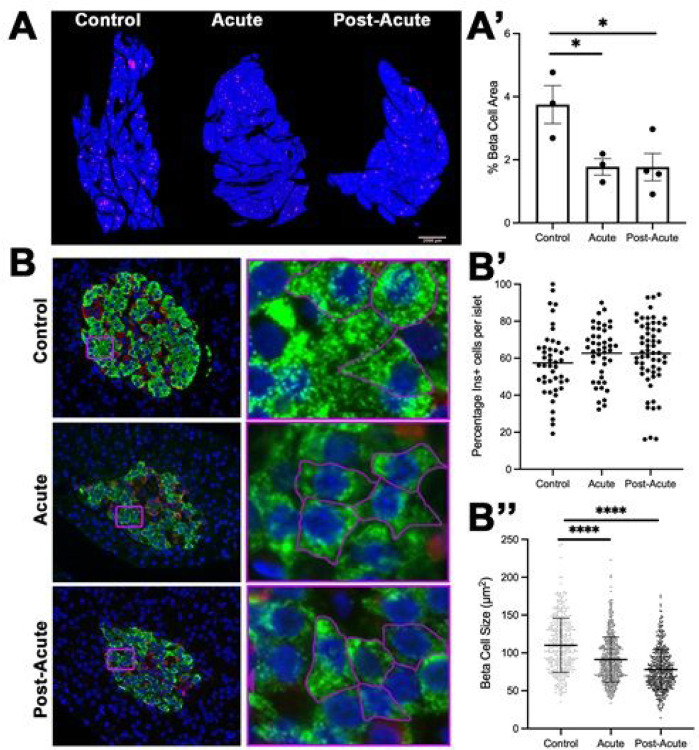
SARS-COV-2 infection induces beta cell atrophy. (A) Representative 5x tile-scan image masks of pancreas stained with insulin (magenta) and DAPI (blue). (A’) Quantification of tile-scan images revealed that pancreas from SARS-COV-2 infected subjects had a 56% decrease in beta cell area (n= 3–4 biological samples per group, *p=0.031 as determined by a one-way ANOVA, *p=0.039 ctrl vs acute by two-tailed t-test, and *p=0.040 ctrl vs post-acute by two- tailed t-test). (B) Representative images of the immunofluorescent staining for beta cells (insulin, green) and alpha cells (glucagon, red) in islets of SARS-CoV2 inoculated monkeys show normal islet composition. Purple inset boxes are small areas magnified to illustrate outline to quantify beta cell size. (B’) NHPs had no statistically significant difference in beta cell composition between time points as determined by a one-way ANOVA (n= 3–4 biological samples per group, each dot represents one islet). (B”) Quantification of beta cell size beta cell size in control and infected pancreata (n=380–529 beta cells measured from 3–4 biological samples per group. p values determined by a one-way ANOVA ****p<0.0001 and unpaired t-test (ctrl vs acute ****p<0.0001), (ctrl vs post-acute ****p<0.0001).

Previous reports suggested that SARS-CoV infection may cause beta cell injury, and other reports have suggested that certain viral infections can cause beta cell loss (30–34). We quantified fractional beta cell area in the control and infected NHP pancreas to determine if SARS-CoV-2 infection resulted in beta cell loss. Tissue was collected from the head, body, and tail of the pancreas and beta cell area was quantified as insulin^+^ pixels divided by total tissue area pixels. Representative images are shown in Figure 2A. Pregnant Zika-infected macaques were excluded from this analysis because pregnancy drives temporary increases in beta cell mass (35, 36). Total beta cell area from acute and post-acute pancreas beta cell area averaged approximately 1.8%, while total beta cell area in control pancreas was approximately 3.8% (n=3–4, p<0.05, Figure 2B). Because it was not clear if the loss in beta cell area was a result of decreased number or decreased size of beta cells (37), we measured the proportion of beta and alpha cells per islet. We found that the percentage of beta cell area per islet did not change (Figure 2C), which suggested that either beta cell atrophy or pan-islet apoptosis could have been driving this phenotype. Neither control, acute, or post-acute beta cells expressed cleaved caspase-3, a marker of apoptosis (data not shown). To measure cellular atrophy, we measured individual beta cell area in each subject (n=300 cells per group). Cellular boundaries were marked with ß-actin staining. We used Image J to trace and calculate individual cell size. Mean beta cell size decreased by 18% in the acute phase when compared to control and by 29% when comparing the post-acute beta cells to those from controls. Within inoculated subjects, individual beta cell size continued to decreased between the acute and the post acute phase, suggesting that SARS-CoV-2 infection continued to drive beta cell atrophy after disease resolution. We measured fasting serum insulin and glucose levels prior to necropsy in a small number of subjects (Supplemental Figure 2). Control subjects (n=2) had very low fasting glucose levels. 4 of 8 inoculated animals had glucose levels 60mg/dL, which has been characterized as dysmetabolic (metabolically normal <60mg/dL, dysmetabolic 60–100mg/dL, diabetic >100mg/dL) (38). Of those 4 animals, 3 also had elevated serum insulin levels (>45mU/ml, dark gray bars).

### SARS-CoV2 infection induces subcellular ultrastructure indicative of diminished beta cell function

Because primates infected with SARS-CoV2 are restricted to BSL3 restricted facilities and these subjects were all participants in other studies, we were not able to pursue *in vivo* beta cell function studies, such as glucose tolerance tests or hyperglycemic/euglycemic clamp studies, to measure how SARS-CoV2 may affect beta cell function. To maximize information we can discern from the tissues we have available, we examined beta cell ultrastructure to assess if the viral infection induced any ultrastructural markers of beta cell stress or dysfunction.

**Figure 3:**
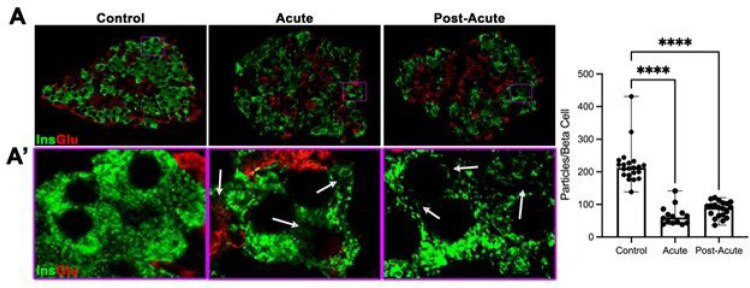
Beta cells from SARS-COV-2 subjects are significantly degranulated. (A) Immunohistochemical staining for insulin and glucagon of representative islets that were imaged using super-resolution microscopy. (A’) High-magnification images purple boxed areas in (A). White arrows highlight large areas in the cytoplasm with reduced insulin staining in pancreas from SCV-inoculated subjects. (B) Quantification of beta cell granulation revealed a greater than 60% decrease in granulation between control and acute/post-acute islets (n=10–20 islets per group from at least 3 different subjects. Each dot represents 1 islet. Two-way ANOVA with Turkey’s multiple comparisons, **** p<0.001)

Close examination of beta cells during individual beta cell size measurement revealed that atrophied beta cells had a degranulated appearance. Degranulation has been proposed to be a driving cause of beta cell deficits in the context of metabolic stress (39, 40). To discern if atrophied beta cells from SARS-CoV2 inoculated pancreas were degranulated, we visualized granular density using super-resolution fluorescence microscopy. Insulin granules were evenly distributed and filled most of the beta cell cytoplasm in control tissues (Figure 3A–A”). In both acute and post-acute beta cells, insulin granules were concentrated in speckles and large areas of the cytoplasm were devoid of insulin granules (Figure 3A–A’, purple boxed insets and white arrows). We used ImageJ to determine the density of granules per square um of insulin area in an islet, then used our previous measurements of beta cell size (Figure 2) to estimate how many granules were present per beta cell. We measured a 66% decrease in insulin granularity between control and inoculated islets (n=10–20 islets per condition, p<0.001). Within the inoculated samples, there was no difference in granularity between the acute and post-acute time points.

**Figure 4:**
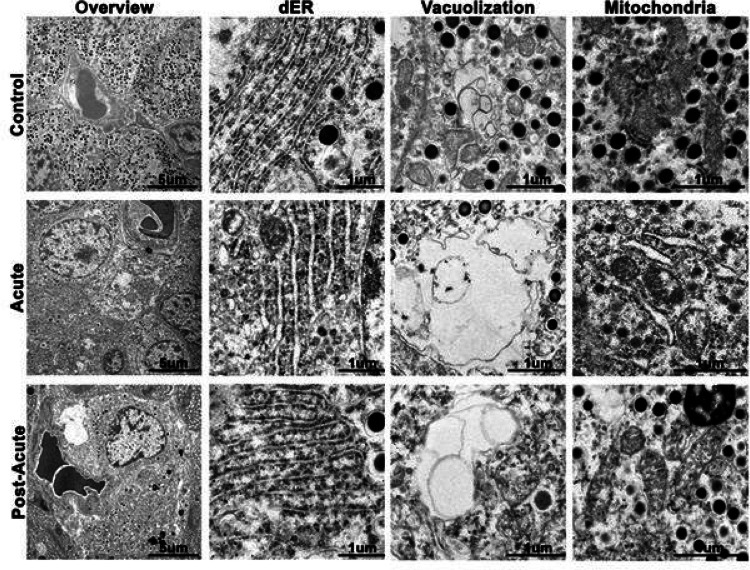
Beta cells from SARS-COV-2 inoculated subjects have ultrastructral hallmarks of beta cell stress. (Overview) Low magnification transmission electron microscopy images highlight multiple cells within islets. Note that the control islet cells contain abundant insulin secretory granules, while acute and post-acute cells are degranulated. (dER) The intraluminal space of the rough endoplasmic reticulum is distended in the acute and post-acute beta cells. (Vacuolization) While cytoplasmic vacuoles are present in control beta cells, vaculoles in the acute and post-acute pancreas are much larger and have internal membranes. (Mitochondria) Mitochondria in the control sample are abundant, large, and have densely packed cristae. Mitochondria from the acute and post-acute pancreas have distended cristae and are frequently ruptured.

To more closely examine the subcellular ultrastructure of the cytoplasm, we imaged control, acute, and post-acute beta cells by transmission electron microscopy. Beta cells from control pancreas exhibited normal ultrastructure, including dense insulin granulation, dense mitochondria, compact endoplasmic reticulum, and minimal vacuolization (Figure 4A). During the acute phase, we detected beta cells that were less electron dense than surrounding cells. These cells had increased vacuole-like spaces, dilated endoplasmic reticulum, and distended cristae within the mitochondria (Figure 4). In less electron dense cells, convoluted membranes predominated the cytoplasms and mitochondria membranes were disrupted. These hallmark attributes mirror observations in beta cells that are undergoing metabolic stress (41–43).

### SARS-CoV2 infection shifts markers of cellular metabolism toward a glycolytic profile

The ultrastructural evidence of beta cell stress, and specifically of mitochondria disruption, raised the possibility these SARS-CoV2 could induce changes in beta cell metabolism. Recent reports concluded that SARS-CoV2 infection in shifts cells towards a more glycolytic metabolism to provide building blocks for viral replication (44). It has been argued that beta cells from Type 2 diabetic patients show a shift in cellular metabolism from oxidative phosphorylation towards glycolysis and that shifts in towards glycolysis can decrease insulin secretion (45). We sought to measure cellular metabolism of beta cells in fixed pancreas from control and SARS-CoV2 inoculated animals.

**Figure 5:**
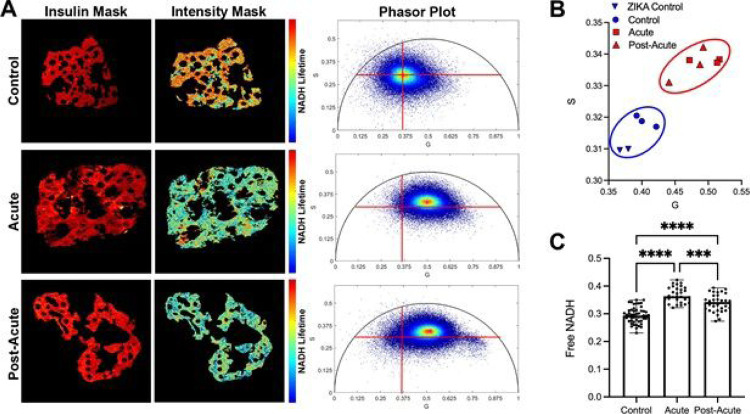
Beta cells from SARS-COV-2-inoculated subjects have a glycolytic metabolic signature. (A) Representative lifetime images of insulin immunohistochemical staining (red mask) and NADH autofluorescence (Intensity mask). In the intensity mask, red pixels indicate a longer NADH lifetime, blue pixels represent a shorter NADH lifetime. Insulin mask was used to filter out the signal from other cell types present in the islets. Beta cell NADH lifetimes were transformed onto phasor plots. (B) The modes of islet phasor plots for each experimental subject were averaged and plotted onto a G vs. S graph. (C) The ratio of free NADH per islet was calculated. N=10 islets per subject, 3 subjects per group).

We developed a novel method to use fluorescence lifetime imaging (FLIM) to measure the levels of NADH in formalin-fixed paraffin embedded beta cells as a proxy measurement of cellular metabolism. FLIM measures the lifetime of excited NADH: unbound NADH exhibits short lifetimes (τ = 0.4 ns) and is a byproduct of glycolysis; enzyme-bound NADH exhibits a far longer lifetime (τ =1.2 – 3.7 ns (46) dependent on the bound enzyme and is a substrate of oxidative phosphorylation (47, 48). This ~10x difference allows FLIM to offer a measure of the glycolytic vs oxidative status of a cell that persists even after fixation and histological processing. We used immunohistochemistry to identify insulin producing cells on slides, then used a 2-photon laser to collect the lifetimes of the secondary antibody for insulin and the autofluorescence of NADH. The distribution of NADH lifetimes within the beta cells of an image is represented on the phasor plot. The closer the phasor plot’s centroid is to the relative position of free NADH on the circle, the higher the glycolytic metabolism of the cell. Figure 5A presents insulin masks, NADH intensity masks, and phasor plots from three representative islets. To capture the glycolytic vs oxidative status of each islet, we averaged the modes of the centroids for each islet per individual pancreas, and plotted the coordinates on a 2D plot (Figure 5B; n=10 islets from 3–5 pancreas per experimental group). We observed that the centroid plots for the acute and post-acute samples clustered separately from the control samples.

We next sought to understand if the separation of the sample populations on the phasor plots represented a change in beta cell metabolism. We calculated each islet’s glycolytic coefficient to report the proportion of NADH from glycolysis and identify cells as primarily glycolytic or primarily oxidative (49). Using this estimation, a higher glycolytic coefficient would represent more free NADH in the islet, suggestive of more glycolytic metabolism. We found that both uninfected control samples and zika-infected samples had a similar glycolytic coefficients (Figure 5C, pregnant- blue triangles, non-pregnant- blue circles). There was a 23% increase in the glycolytic coefficient in islets from the acute pancreas, suggesting that these cells employed a more glycolytic metabolism. Islets from the post-acute pancreas had a slightly lower glycolytic coefficient that was still significantly different from control samples. This indicates that beta cell metabolism may begin to recover in the post-acute phase of COVID19 pathogenesis. Because our study ended 14 days after infection, we were unable to measure if and when beta cell metabolism could return to baseline.

## Discussion

Because the number of patients who have recovered by COVID19 continues to rise, it is imperative to understand if SARS-CoV2 infection causes cellular disfunction that may compromise the long-term health of survivors. Since the beginning of the COVID19 pandemic, commentaries, case reports, and primary data have driven speculation about the possibility of SARS-CoV2 causing a direct or indirect injury to pancreatic beta cells. This has been difficult to address in affected patients because of the inaccessibility of living human pancreatic tissue; it is also difficult to assess in human autopsy samples because of poor tissue quality due to post-mortem autolysis. To address this controversy, we have interrogated the pancreas from a rhesus macaque model of COVID19 pathogenesis that mirrors mild to moderate human COVID19 disease progression, which accounts for the vast majority of all COVID19 infections. Because primates are the closes relatives to humans, this model has the advantage of being a system that reflects human disease progression better than other animals. It also more accurately reflects the severity of most COVID19 cases, thus being an ideal model for understanding how COVID19 may affect the broad spectrum of patients with the disease, not just the most severely ill.

We demonstrated that SARS-CoV-2 can be detected in pancreatic beta cells after intranasal and intratracheal inoculation with the virus, therefore, SARS-CoV-2 can infect pancreatic beta cells *in vivo*. This is consistent with reports of SARS-CoV-2 infecting human islets in vitro and autopsy samples (9, 50–52), but in conflict with other reports that argue that the canonical receptors for SARS-CoV2 expression are not expressed in human islets (6, 7). After extensive measurements of beta cell area, islet composition, and individual beta cell size, we also concluded that beta cell atrophy accounts for a decrease in beta cell area. Super-resolution and ultrastructural analysis indicated that beta cells are degranulated and displayed hallmark signs of beta cell stress.

It was reported that SARS-CoV2 infection can shift cellular metabolism towards glycolysis as a means of making metabolites available to support viral replication (44). It has also been reported that increased glycolytic metabolism decreases insulin secretion (45). We used a novel approach to FLIM to assess if SARS-CoV2 infection shifted beta cell metabolism towards a glycolytic profile. The quantitative analysis of FLIM imaging determined that there are higher amounts of free NADH in the islets of inoculated subjects, thus suggesting a more glycolytic metabolic profile. Our data documents a minor but significant decrease in NADH levels during the post-acute period, suggesting that beta cell metabolism may be able to recover over time. This data, coupled with evidence of direct infection of beta cells by the virus, support the conclusion that SARS-CoV2 has a direct effect on beta cell function.

One limitation of this study is our inability to test beta cell function after SARS-CoV2 inoculation to measure the impact of viral infection on beta cell function directly. Because these subjects were part of ongoing studies for pre-clinical pharmaceutical trials, we were unable to perform glucose tolerance tests and measure insulin secretion. Nonetheless, previously published reports have determined that *in vitro* infection of islets with SARS-CoV2 decreases glucose stimulated insulin secretion. Our reporting of beta cell atrophy, beta cell degranulation, and disruption of subcellular beta cell ultrastructure are shared with and supported by the studies that have reported beta cell dysfunction after SARS-CoV2 infection in vitro (50).

As millions of patients have recovered from COVID19, it is critical to understand if beta cells were injured by SARS-CoV2 infection and if they recover from injury. It is documented that patients with Type 2 diabetes have elevated insulin requirements during hospitalization for COVID19 and that hyperglycemia is a comorbidity of COVID19 infection. Our own group has recently reported a concerning spike in children presenting with new onset type 2 diabetes in diabetic ketoacidosis during the COVID19 pandemic. Ketoacidosis can be a clinical indicator of acute beta cell loss of function (53). Our data shows that SARS-CoV2 infection can induce beta cell stress, which generally leads to beta cell dysfunction, and it is not clear that beta cells recover from that stress in the immediate post-acute phase after disease resolution. Further studies are required to understand if that beta cells can recover after the initial injury from SARS-CoV2 infection, which could have implications for the future health of millions who have recovered from COVID19.

## Materials And Method

All experiments using tissues from SARS-CoV2-inoculated or ZIKA-infected subjects were inactivated with 4% formaldehyde. All experiments were approved by Children’s Hospital Los Angeles Biosafety committee. All animal studies were conducted in compliance with all relevant local, state, and federal regulations and were approved by the Bioqual Institutional Animal Care and Use Committee (IACUC).

### Animals and Study Design

Rhesus Macaque model: Non-human primate models consisted of thirteen outbred, Indian-origin adult (between 3–7 years old) male rhesus macaques (Macaca mulatta) housed at Bioqual, Inc (Rockville, MD). The rhesus macaques were randomly stratified into three groups of three animals each. The first study inoculated nine animals with SARS-CoV-2 with a total 1mL dose of 1.1×10^6^ plaque forming units (PFU) The 1 mL doses were administered via either the intranasal (0.5 mL per nostril) or intratracheal routes. Animals were then observed for signs of disease. Veterinary staff performed semi-quantitative clinical assessments based on four categories: clinical appearance, dyspnea, recumbency, and responsiveness. Animals were then assigned for necropsies on days 7–10 (acute, N=3) or day 14 (post-acute, N=4) post-inoculation.

SARS-CoV-2 stock: The SARS-CoV-2 USA-WA1/2020 stock from the BEI Resource (NR-42281; Lot 370033175; courtesy of Natalie Thornburg, Centers for Disease Control) was used and propagated on Vero E6 cells. The viral challenge stock was then harvested on day 5 post infection at 90% cytopathic effect (CPE). Whole-genome sequencing confirmed 100% identity with the parent virus sequence (GenBank MN985325.1; courtesy David O’Connor, Shelby O’Connor, University of Wisconsin).

### Histology

Pancreata were cut into head, mid, and tail sections and embedded into paraffin blocks. Blocks were sectioned into 7 micron slices on charged slides.

Slides were stained as previously described with the exception of the ACE2 and Nucleocapsid stains, which were performed using a pressure cooker and citrate buffer for unmasking rather than a microwave and citrate buffer. All slides were and mounted with ProLong Diamond Antifade (Thermo Fisher cat# P36961). The complete antibody list can be found in supplementary table 1.

### In-situ Hybridization and co-IF

We followed the RNAscope® Multiplex Fluorescent Reagent Kit v2 (ACDbio) protocol for paraffin sections. Protease plus for 20 mins. 845701 RNAscope® Probe - V-nCoV2019-S-sense. Immediately following the opal secondary steps, we incubated slides in blocking buffer (BSA, tween, tbs) for 1 hour, followed by overnight insulin staining. The next day, slides were washed and incubated with a secondary antibody and DAPI, washed, and mounted.

### Transmission Electron Microscopy (TEM) Sample Preparation and Imaging

Pancreatic beta-cell ultrastructure was imaged using the Talos TEM. Briefly, pancreatic tissue samples (control, acute, and post-acute) were in 4% paraformaldehyde in PBS, then in 2.5% glutaraldehyde and 2% paraformaldehyde in 0.1M HEPES and postfixed in 1% osmium tetroxide overnight. The fixed samples were stained with 1% uranyl acetate for an hour and dehydrated with an increasing percentage of ethanol solutions. Propylene oxide (PO) was used as a transition fluid and embedded with a medium resin hardness using the Embed 812 kit (EMS) which polymerized at 60 degrees Celsius for a minimum of 18 hours. Ultrathin sections (80nm) were obtained using a Leica UC6 ultramicrotome. Once mounted on grids, sections were treated with 3% H2O2, then stained with lead citrate followed by uranyl acetate. The stained sections were examined using Talos F200C TEM operated at 80kV. Images were taken with a mounted Ceta Camera. N=2–4 biological samples per condition.

### Microscopy

Fluorescence images were acquired with a DM4000B microscope equipped with 20x/0.7 HC PL APO and 40x/0.85 HCX PL APO objective lenses and DFC360 FX camera (Leica Microsystems, Buffalo Grove, IL). Fluorescence excitation and emission bands were as follows: 360/40 and 470/40 nm for DAPI; 480/80 and 527/60 nm for Alexa Fluor 488; 546/12 and 600/40 nm for Cy3; and 620/60 and 700/76 for Alexa Fluor 647. Pixel sizes were 0.323 μm for 20x and 0.161 μm for 40x images. The system was controlled with LAS X 3.6 software.

Confocal images were acquired with an LSM 710 system mounted on an AxioObserver.Z1 microscope equipped with 20x/0.8 Plan-APOCHROMAT and 63x/1.4 oil Plan-APOCHROMAT objective lenses (Carl Zeiss Microscopy, White Plains, NY). Fluorescence excitation lasers and emission detection ranges were as follows: 405 nm/406–480 nm for DAPI; 488 nm/490–550 nm for Alexa Flour 488; and 555 nm/560–620 nm for Cy3. Voxel sizes were 0.3 × 0.3 × 1.0 μm for 20x and 0.1 × 0.1 × 0.3 μm for 63x images. The system was controlled by ZEN 2011 software.

Fluorescence Lifetime Imaging Microscopy (FLIM) was performed on SP8 DIVE FALCON spectral multi-photon FLIM microscope (Leica Microsystems, Germany) using 40x/1.10 N.A. water immersion objective. NAD(P)H was excited with a Spectra-Physics Insight 3X ultrafast IR laser at 740 nm, 0.8mW average power, and 4 frame accumulations per optical section. The Alexa dyes were excited using 860nm wavelength with the same Spectra-Physics laser. Images were collected at 1024 × 1024 resolution and 2.0 zoom. N= 3–5 biological samples and >14 islets per condition.

### Super Resolution Imaging and Processing

Slides were stained as previously described with monoclonal rabbit anti-glucagon (Abcam, ab92517) and polyclonal guinea pig anti-insulin (Dako, A0564) for primary antibody incubation and polyclonal Goat AF594 anti-rabbit and donkey AF647 anti-guinea pig, respectively (ThermoFisher, A-11012; Abcam, ab150187) all at 1:500 dilution. They were then imaged on a Leica Stellaris 8 Confocal Microscope with an integrated Lightning detection using a 67x oil immersion objective. The Alexa dyes were excited at 593nm and 647nm. Images were collected at 5192 × 5192 and 0.75 zoom. Insulin stained (AF647) images were then processed on Fiji ImageJ using the “Find Maxima” function with a prominence set to greater than 10 to obtain the count of particles of green signal within the image. Next, the threshold for the images were adjusted to capture the insulin positive cells. With the new binary image, the pixels were dilated with 50 iterations and 4 counts. Areas of the insulin stained parts of the image were measured using the “Analyze Particles” function with the size (μm2) set at 0-Infinity and circularity from 0.00–1.000. The results are summarized with the total amount of particles and their combined total area. The average size of a beta cell nucleus (6.9μm2, Saisho et al. 2013) was subtracted from the average beta cell size obtained from the previously mentioned analysis to acquire beta cell cytoplasmic area for each condition (control=103.217, acute=84.3932, and post-acute=71.0426). To obtain the final unit of particles per beta-cell, the count of particles was divided by the cytoplasmic area. One-way ANOVA was used to determine significant differences between each condition.

### Image Analysis

#### Islet Composition:

We imaged a minimum of 10 islets per biological sample, with 3–4 biological samples per treatment group. Statistical significance was determined using an ordinary one-way ANOVA.

#### Beta-Cell Area:

Tile scanned 5x images were stitched and analyzed in FIJI/ImageJ for analysis. Images were cropped using the freehand selections tool to exclude lymph nodes and blood vessels. We then set the image to binary and dilate settings before measuring the area for the DAPI and Insulin channels separately. We then divided the insulin channel area by the DAPI channel area and multiplied by 100 to get Beta-cell mass percentage. N=3–4.

#### Beta-Cell Size:

Islets stained for beta-actin and Insulin were imaged at 20x and analyzed in FIJI/ImageJ. We used the freehand selections tool to follow the beta-actin outline of individual cells in an islet and used the fill tool to fill in the cells. We then measured the area of the filled in cells. N=3–4 biological samples, with over 380 individual beta cells measured per timepoint. Statistical significance was determined using an ordinary one-way ANOVA.

### FLIM Processing

To analyze the metabolic signature for each cell type in the islet, masks for regions of pancreatic alpha cells and pancreatic beta cells are created from the microscopic images of the GLUC and INS staining respectively by thresholding. masks were then preprocessed to fill the cytoplasms of cells and exclude the nucleus. Each mask was applied to the field of view to extract lifetime information from beta cells and alpha cells separately. We used the mode of the resulting phasor clusters to represent the sample to minimize the effect of contaminating fluorescent species such as lipofuscin. We calculate the glycolytic coefficient and the major contributing enzyme by drawing a line from the phasor position representing free NADH (0.4 ns) through each cluster’s mode and extrapolate to an intersection with the universal circle. We refer to this line as a metabolic trendline. The glycolytic coefficient falls out from the linear properties of the phasor; the fractional distance of the mode along the trendline chord (all metabolic trendlines have a length of 1). The closer the mode is to the free NADH phasor position, the higher the glycolytic coefficient. Major contributing enzymes are determined based on known lifetimes at the extrapolated ends of each metabolic trendline intersecting the universal circle. One-way ANOVA was used to determine significant differences between each condition. N= 3–5 biological samples and >30 islets per condition.

## Supplementary Material

Supplement 1
