# Supplementary material for: SARS-CoV2 infects pancreatic beta cells in vivo and induces cellular and subcellular disruptions that reflect beta cell dysfunction
Source: Res Sq. 2021 Jul 20:rs.3.rs-592374. Preprint. [Version 1] doi: 10.21203/rs.3.rs-592374/v1 (PMC8312902; doi:10.21203/rs.3.rs-592374/v1)
Supplement: Supplement 1 [file e21ac022d23f2f9f398d7394.pdf]

Supplemental Figure 1

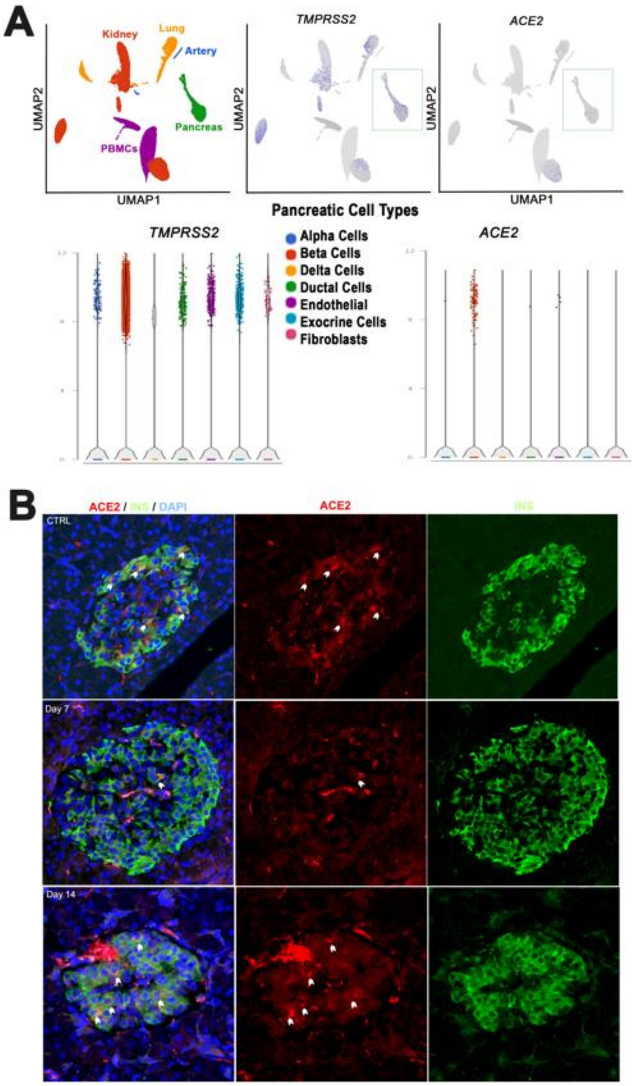

Supplemental Figure 1: ACE2 expression in the *Rhesus Macaque* pancreas. (A) Single cell RNA-seq analysis shows that both *TMPRSS2* and *ACE2* are expressed in pancreatic cell types. *TMPRSS2* and *ACE2* transcript expression were assigned to the pancreatic cell subtypes, as identified by cell-type specific marker expression. **(B)** Representative images of the immunofluorescent staining for ACE2 (red) and Insulin (green) in control, acute, and post-acute pancreas. White arrows denote beta cells with increased ACE2 expression.

Supplemental Figure 2

Sample #	Fasting Serum Glucose (mg/dL)	Fasting Serum Insulin (mU/mL)
MK305.3	26	133.5
MK305.4	27	82.1
MK1084.5	46	15.6
MK1084.3	49	36.6
MK1084.4	49	17.5
MK1084.7	52	77.8
MK1084.8	60	16.9
MK1084.1	60	60.9
MK1084.6	70	81.8
MK1084.2	77	92.9

Supplemental Figure 2: Fasting glucose and insulin measurements for control and post-acute subjects. Serum samples taken immediately prior to necropsy were used to measure glucose and insulin. MK305.3 and MK 305.4 were control samples, all others were inoculated with SARS-COV-2 14 days prior to necropsy.
